# Supranuclear eye movements and nystagmus in children: A review of the literature and guide to clinical examination, interpretation of findings and age-appropriate norms

**DOI:** 10.1038/s41433-018-0216-y

**Published:** 2018-10-23

**Authors:** D. Osborne, M. Theodorou, H. Lee, M. Ranger, M. Hedley-Lewis, F. Shawkat, C. M. Harris, J. E. Self

**Affiliations:** 1 University Hospital Southampton, Southampton, UK; 2 Moorfields Eye Hospital, London, UK; 3 Clinical and Experimental Sciences, University of Southampton, Southampton, UK; 4 University of Plymouth, Plymouth, United Kingdom

**Keywords:** Paediatrics, Eye manifestations, Neuromuscular disease

## Abstract

Abnormal eye movements in children, including nystagmus, present a significant challenge to ophthalmologists and other healthcare professionals. Similarly, examination of supranuclear eye movements and nystagmus in children and interpretation of any resulting clinical signs can seem very complex. A structured assessment is often lacking although in many cases, simple clinical observations, combined with a basic understanding of the underlying neurology, can hold the key to clinical diagnosis. As the range of underlying diagnoses for children with abnormal eye movements is broad, recognising clinical patterns and understanding their neurological basis is also imperative for ongoing management. Here, we present a review and best practice guide for a structured, methodical clinical examination of supranuclear eye movements and nystagmus in children, a guide to clinical interpretation and age-appropriate norms. We also detail the more common specific clinical findings and how they should be interpreted and used to guide further management. In summary, this review will encourage clinicians to combine a structured assessment and a logical interpretation of the resulting clinical signs, in order to recognise patterns of presentation and avoid unnecessary investigations and protracted delays in diagnosis and clinical care.

## 摘要

目前, 儿童眼球运动障碍已成为眼科医生和医疗保健人员的重大挑战。同样, 对于儿童核上性病变眼球运动的检查和对其产生的相应临床体征的解释也非常复杂。核上性眼球运动障碍主要表现为对称性眼球垂直运动障碍。尽管在许多情况下, 缺乏对疾病系统性评估, 但是简单的临床观察结合对神经病学的初步认识, 仍是临床诊断的关键。由于儿童眼球运动障碍的初步诊断范畴广范, 认识临床分型和了解其神经病学机制对于目前疾病的管理必不可少。在此, 我们提出了针对儿童核上性眼球障碍的系统性、有条理的临床检查综述和最佳实践指南, 适用于不同年龄段核上性眼球运动障碍的患儿。我们还详细介绍了更常见的特定临床发现, 以及如何解释并使用它们进一步指导进行疾病的管理。总之, 这篇综述将指导临床医生将系统性评估病情和临床体征的合理解释结合起来, 以识别临床分型, 避免过度检查和误诊。

## Introduction

The function of eye movements is to bring visual stimuli to the fovea and hold them there, during head movements or movement of the stimuli themselves. Examination of these eye movements in children, in an outpatient setting, can present a clinical challenge. However, the information gained from an effective examination can help the clinician discern benign from pathological disease, localise neuropathology and non-invasively monitor a variety of neurological disorders. Hereafter, we outline methods of eye movement examination, the most important findings and their clinical implications for clinicians without access to a dedicated eye movement laboratory.

Human supranuclear eye movement pathways can be conceptualised as providing solutions to the following visual demands: to compensate for large head movements (the vestibulo-ocular reflex (VOR)), to use visual information to fine-tune the VOR and to return the eyes to the central position after full excursion (the optokinetic reflex (OKR)), to track a moving visual target without moving the head (Smooth Pursuit, SP), to place a visual target onto the fovea (saccades) to maintain eccentric eye position (gaze holding) and to allow binocular co-ordinated eye movements to achieve and maintain binocular foveation (vergence movements). Table 1 summarises the supranuclear eye movement control systems of greatest clinical relevance in humans.

**Table 1:** A summary of human supranuclear eye movements and their evolutionary prompts [1, 29, 30]

Evolution		
Evolutionary prompt	Type of eye movement	Function
Balance organs (such as the semi-circular canals) evolve and mature. Maintaining retinal image stability during self-motion becomes an advantage	Vesibulo-ocular movements (the slow component of the VOR reflex)	Maintain eye position during head movements regardless of fixation
Visual advantage of fine tuning gross VOR movements and repositioning the eyes quickly after full excursion (to minimise retinal blur)	Optokinetic reflex (OKR)	To maintain eye position and fine-tune VOR responses using visual proprioception during body movement or when viewing a moving visual scene
Evolution of a retinal area of higher visual acuity (e.g., fovea)	Saccades	Fast movements to permit re-fixation of targets of interest onto the foveae (retinal areas of maximum acuity)
Evolution of a retinal area of higher visual acuity (e.g., fovea)	Gaze holding	Tonic stimulation of extra-ocular muscles to keep the eyes in eccentric gaze
Large visual fields become less of an advantage than stereovision. Eyes become frontal and retinal correspondence develops as a prerequisite for stereovision	Binocular vergence movements	To coordinate convergence and divergence and permit foveation and stereovision
Frontal eyes and fovea mean that binocular tracking of moving visual targets improves vision	Smooth pursuit (SP)	Binocular, co-ordinated smooth tracking of visual targets

## History taking

As with all clinical assessments in children, a detailed history from the parent and child is important and can direct the clinician to certain aspects of the subsequent examination. Table 2 details some of the most useful questions to be included in a thorough history from a child with an eye movement disorder.

**Table 2:** Questions which can direct the clinician prior to examining a child with abnormal eye movements

Question	Clinical relevance
Pregnancy, maternal medication/drug use and birth history	Maternal drug exposure and prematurity are associated with an array of eye movement abnormalities
Family history of eye/neurological disease/systemic disease	Many eye movement disorders have a hereditary component with different inheritance patterns indicating which genes may be involved. Neurological symptoms in relatives can also suggest an underlying aetiology (e.g., Spinocerebellar ataxias) [31]
Specific questions about visual behaviours—e.g., nyctalopia or photophobia	Photophobia and nystagmus are common findings in disorders of cone function and albinism. High frequency nystagmus with photophobia is more common in cone dysfunction. Nyctalopia is a common symptom in rod dysfunction
Does the child blink excessively or head thrust towards direction of intended gaze?	Can be seen in Saccadic initiation failure (SIF)
Open questioning about other visual behaviours	Parents will often report a very detailed description of visual behaviours, which can direct clinical examination such as a child with chin depression and vertically ‘wobbly eyes’ (commonly seen in down beat nystagmus), or pushing/rubbing eyes firmly for retinal stimulation in blind babies/children
Does the child experience oscillopsia?	Lack of oscillopsia in the presence of involuntary eye movements such as nystagmus, suggests early-onset; constant oscillopsia suggests an acquired disorder
If oscillopsia is reported, is it when stationary or when moving?	Oscillopsia which is only present during head movement implies a vestibular pathology [32]
Are there associated speech or swallowing problems?	Possible brainstem pathology or Myasthenia Gravis
Are there associated coordination problems?	Possible cerebellar pathology
Is there associated hearing loss or tinnitus?	Possible peripheral vestibular pathology
Is the patient on any medications?	Many medications can cause abnormalities of eye movement, most commonly anti-epileptic medication
Are there any concerns about any other aspect of the child’s development or health besides their eyes?	Eye movement abnormalities form a part of many multisystem syndromes and can be the presenting feature

## Clinical examination

When examining supranuclear eye movements in a child, it is important to assess each separate eye movement control system in a systematic way, with an understanding of what to expect [1]. ‘Normal findings’ will vary with age. Table 3 summarises some of the normal clinical findings in infants and children using more freely available clinical equipment (such as the OKR drum rather than a full-field OKR stimulus). Subsequently, we describe clinical examination techniques for each system.

**Table 3:** A summary of normal clinical findings when examining supranuclear eye movements in children [1, 33, 34]

Age	Eye movement					
	VOR	OKR (or OKN)	Saccades	Gaze holding	Vergence	Smooth pursuit
Full-term infant	Both slow and quick phases are present in most. ‘Locking up’ (eyes fixed in either left or right gaze) due to lack of the quick phase can be seen in some normal infants until 45 wks gestation	Binocular OKR is present. Monocular OKR is present to temporal-to-nasal but not nasal-to-temporal stimuli. Approximately 1–2 fast phases per second	Saccades are hypometric (fall short of target). Small secondary saccades can be seen especially after large saccades. Appear to have normal speed	Very eccentric gaze holding is rarely seen in healthy neonates. To moderate eccentricities, it appears normal (no back-drift)	Most are slightly divergent and no convergence movements are seen	Not usually present. Coarse, often jerky (saccadic) movements to large, slow targets develop in first few weeks
3 months	Clinically normal	Naso-temporal asymmetry (described above) disappears to moderate stimulus speed	Saccades become less hypometric and secondary saccades are smaller but may be seen	As above	Divergence has reduced or gone and coarse convergence movements can be seen	Usually seen to large slow moving targets. Become saccadic if target is moved more quickly
6 months	Clinically normal	As above	As above	Clinically normal	Divergence has usually gone and convergence movements are more established as fusion develops	Start to become saccadic only to fast target speeds
1 y	Clinically normal	Clinically normal with fast phase frequency increasing to 2–4 per second	Clinically normal (still hypometric as in adults but secondary saccades rarely seen)	Clinically normal	Clinically normal	As above
5 y	Clinically normal	Clinically normal	Clinically normal	Clinically normal	Clinically normal	Clinically normal

## Vestibulo-ocular reflex (VOR)

### Underlying neuroanatomy

**Fig. 1:**
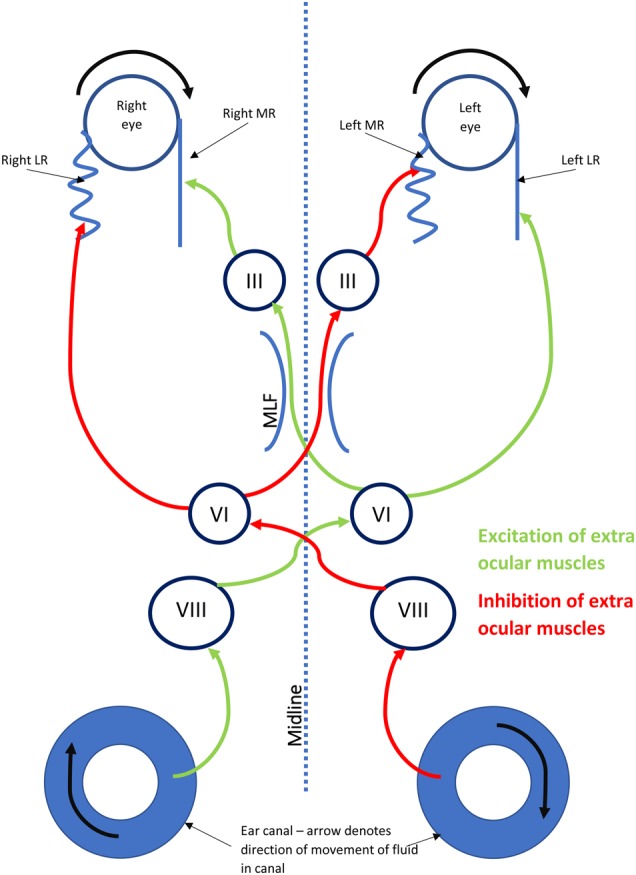
The vestibulo-ocular reflex (VOR). LR lateral rectus; MR medial rectus; MLF medial longitudinal fasciculus; VI sixth nerve nucleus; VIII 8th nerve (vestibular) nuclei; III third nerve nucleus. This diagram represents a right face turn head movement with eye movement to the left

A three-neuron arc, including the vestibular ganglion, vestibular nuclei and the oculomotor nuclei (Fig. 1) [2] operate the VOR system, which holds the eye still in space during head movements. During head motion, movement of the semi-circular canals relative to endolymph creates an afferent signal, which passes via the vestibular nerve to the ipsilateral vestibular ganglion in the internal auditory meatus. These signals are relayed to the ipsilateral vestibular nuclei (Pons and Medulla), which then transmit excitatory inputs to the contralateral oculomotor nuclei, and inhibitory inputs to the ipsilateral oculomotor nuclei (Midbrain and Pons). The oculomotor nuclei pass motor signals to the yoked extra-ocular muscles via the IIIrd, IVth and VIth cranial nerves.

#### Clinical assessment of the VOR

A mismatch between the speed of head movement and the speed of the VOR movement (VOR gain) can be tested in older children by measuring visual acuity during slow head movements (which should not change). However, this test is rarely used in the clinic as it is not possible in preliterate children and isolated abnormalities of VOR gain are rare.

‘Dolls Head Manoeuvre’ (Oculocephalic Manoeuvre) or Spinning baby tests are the standard tests for VOR in the clinic.

##### Doll’s head manoeuvre

**Fig. 2:**
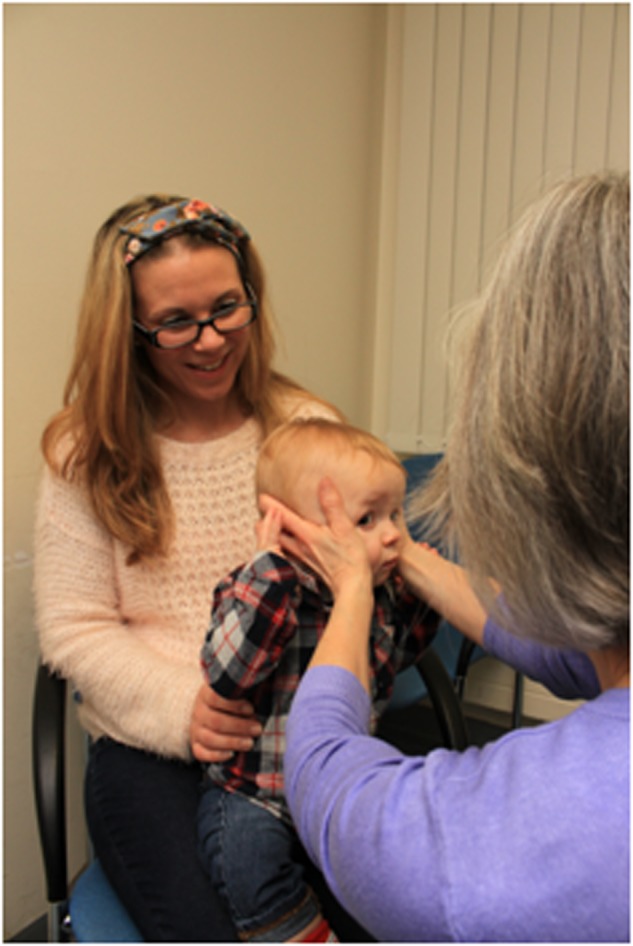
How to perform the doll’s head manoeuvre on an infant in a clinical setting

The infant’s head is held with both hands and rapidly, but gently, moved horizontally and vertically to test both VOR, respectively. The eyes should stay fixed in position despite the movements. The rotations can be small and should not be performed in children with cervical spine problems or downbeat nystagmus (Fig. 2).

##### Spinning baby test

**Fig. 3:**
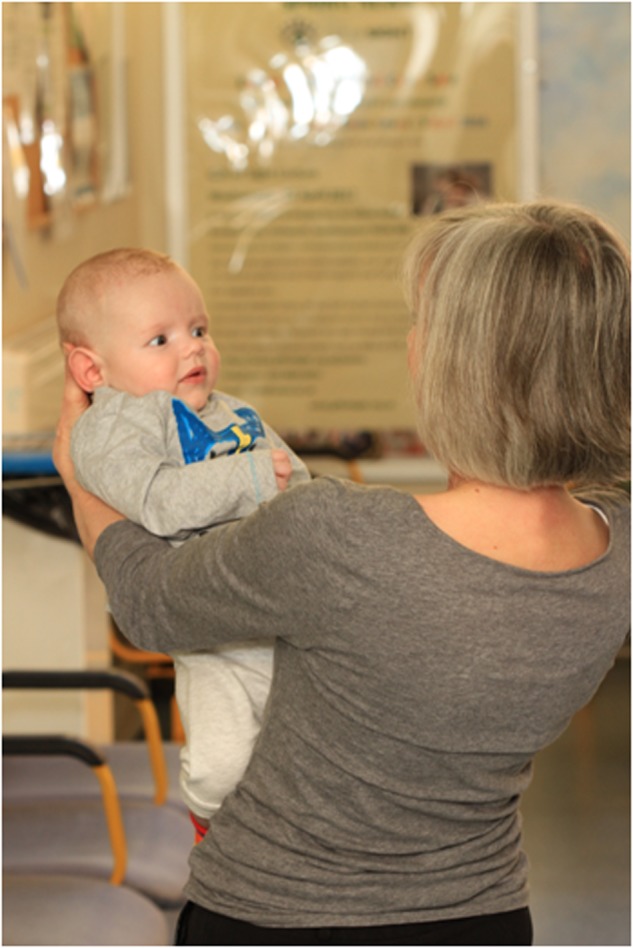
Performing the spinning baby test in a clinical setting

The examiner stands and holds the infant at arm’s length. In the case of a very young infant the back of the head is supported as shown. The examiner rotates to the right with the infant through 2–3 revolutions, whilst observing the infant’s eyes. This induces a per-rotatory nystagmus (driven by VOR and OKR) in the infant with fast phases to the examiners left. The rotation is then abruptly stopped inducing a post-rotatory vestibular nystagmus in the infant, with fast phases beating to the examiners right. The procedure is repeated in the opposite direction and in small infants can also be used to test vertical VOR by laying the infant flat in the examiners arms. In older children this test can be modified by the child sitting on an examiners lap and facing them while both rotate on a swivel chair. Older children can also be examined by sitting alone in a swivel chair and spun. Normal findings include a large per-rotatory nystagmus (unless the child is fixing on the examiners face) but importantly, only two to three post-rotatory nystagmus beats in the opposite direction (Fig. 3).

### Interpreting some common findings during VOR testing

- Loss of the fast phase of VOR, can be a normal finding in infants before 45-weeks gestation or reflect a saccade abnormality, for example, Saccadic initiation failure (SIF). This can be seen as ‘Locking up’ during spinning (i.e., both eyes fixed at the limit of gaze in the opposite direction to spinning [3]).
- During doll’s head testing, a catch-up saccade, when testing in one direction only, suggests a specific semi-circular canal pathology.
- In the presence of acute vertigo in older children, abnormal VOR in one direction, lack of a skew deviation and unidirectional nystagmus suggest a peripheral vestibular (e.g., vestibular neuritis) rather than central cause (e.g., stroke).
- After spinning, post-rotational nystagmus lasting more than two to three beats is a sign that the VOR is not being supressed by visually guided reflexes. This is either an indication of very poor vision or a cerebellar disorder (e.g., ataxia-telangiectasia [4]).
- The VOR reflex may be decreased or absent in children with the CHARGE syndrome (a disorder characterised by coloboma, heart defects, atresia choanae, growth retardation, genital abnormalities and ear abnormalities) [5], in type 1 ushers syndrome [6] or forms of inherited ataxia (e.g., spinocerebellar ataxia type 3, Friedreich’s ataxia [4]).

## OKR (also called OKN)

### Underlying neuroanatomy

Some authors consider the pathway for OKR, tested with an OKR drum or tape in the clinic, as the same as the smooth pursuit pathway, involving striate and extra-striate cortices with descending connections via the internal capsule to the brainstem pontine nuclei, cerebellum (floccular region), vestibular nuclei and the oculomotor nuclei [7]. Most clinicians do not have access to testing methods other than a hand-held tape or drum, therefore, the following information is directed towards findings elicited by these tests.

### Clinical assessment of the OKR

**Fig. 4:**
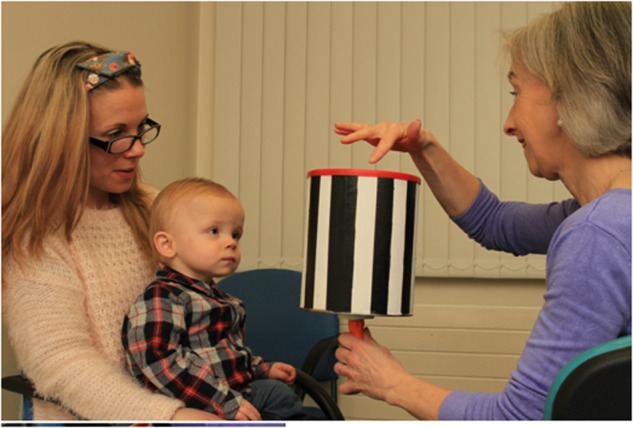
Using an OKR Drum in a clinical setting

*OKR drum or tape* is held at 50 cm from the child’s face and rotated slowly in both horizontal and vertical directions whilst observing the child’s eye movements. It is performed both monocularly and binocularly. The examiner would expect to see slow eye movements in the direction of the stimulus with a fast movement seen in the opposite direction (Fig. 4).

### Interpreting some common clinical findings during OKR testing

- Lack of any OKR with drum or tape testing can be a sign of either extremely poor vision or delayed visual maturation (DVM), but may also be seen in drowsy infants. Interestingly, a full-field OKR can be elicited in some patients with DVM even where a small field OKR is absent. It has been hypothesised that using a full-field stimulus may elicit an OKR reflecting a more primitive, subcortical OKR subtype called OKNd [8], however, this test is not freely available.
- Vertical OKR can be demonstrated in young infants with horizontal nystagmus as a superimposed waveform if there is moderate vision. It can be used (prior to EDTs) to reassure that there is unlikely a profound visual disorder. There has been debate about whether horizontal OKR is lost, reversed or altered by shifting null zones in congenital nystagmus. By clinical observation alone, it is rare to observe robust horizontal OKR in the presence of horizontal nystagmus but where clearly demonstrable, possibly suggests that an ‘idiopathic’ or albinism related cause is less likely.
- Quick phases of the OKR are intermittently absent in SIF (see disorders of saccades below). Similar to VOR testing, it can result in a lack of OKR or in deviation of the eyes in the direction of the slow phases because of a mismatch between the slow phase movement and the insufficient corrective saccade.
- Asymmetry in response to a right vs left moving OKR stimulus, when tested binocularly, indicates a unilateral cortical or pontine lesion. Asymmetry of OKR when tested in this way may occur in some patients with early-onset poor vision in one eye.
- A vertical binocular OKR response which is worse than horizontal response indicates a midbrain lesion.

## Saccades

### Underlying neuroanatomy

**Fig. 5:**
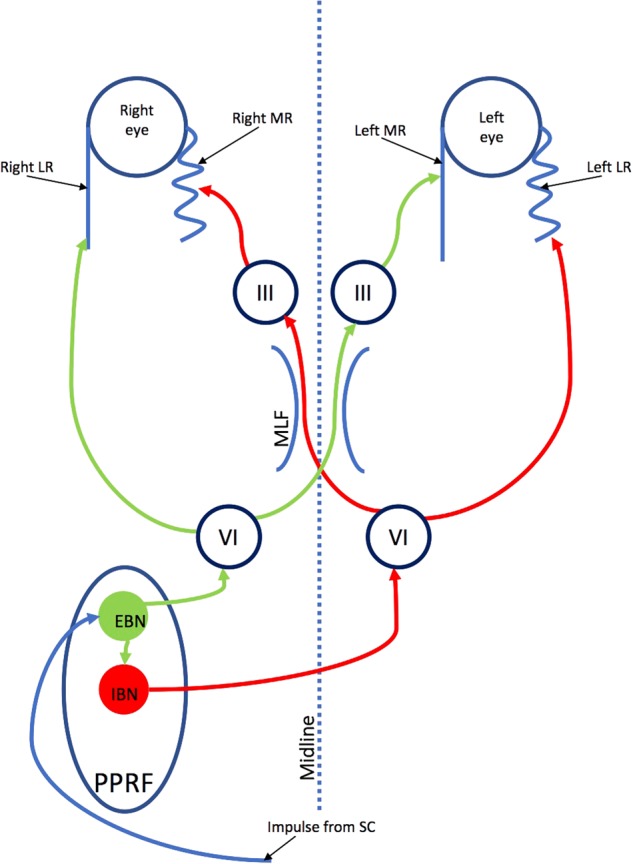
Saccades. LR lateral rectus; MR medial rectus; MLF medial longitudinal fosiculus; EBN excitory burst neurons; IBN inhibitory burst neurons; SC superior colliculus; PPRF pontine paramedian reticular formations. This diagram shows the pathway through the midbrain/pons of a saccade to the patient right

Saccades are fast, voluntary or involuntary eye movements initiated in the visual cortex under the control of frontal oculomotor areas for voluntary saccades and the parietal cortex for reflexive saccades. The pathways then project through the internal capsule, divide into a dorsal limb which projects to the superior colliculus and a ventral limb (oculomotor pathways) which projects to the pons and midbrain nuclei. In the pons and midbrain, the neurons from the ventral limb input into a local control system for saccadic firing. This comprises excitatory burst neurons (EBNs), which fire to generate saccades, inhibitory burst neurons (IBNs), which are activated by EBNs and inhibit firing of neurons to contralateral extra-ocular muscles and pause units. IBNs tonically fire to inhibit the EBNs and therefore continuously inhibit spontaneous saccades. Horizontal saccades are mediated by the ipsilateral pons and contralateral frontal eye field (FEF) and parietal eye field (PEF); vertical saccades are generated in the midbrain. The EBNs project to the ipsilateral 6^th^ nerve nucleus as well as contralateral third nerve nucleus via the medial longitudinal fasciculus (MLF) (Fig. 5).

From a clinical stand point, it is important to remember that horizontal and vertical saccades are localised separately in the ponto-medullary junction and the midbrain respectively and that the cerebellum controls the metrics for both.

### Clinical assessment of Saccades

Clinically, saccades can be tested in many ways according to age. The previously described *spinning baby test*, includes a saccadic component and can be the most informative assessment in younger infants. In older children, a brightly coloured target is presented in front of the child at a large angle (with or without a simultaneous sound in a neutral position) and eye movements are simply observed for direction, accuracy and symmetry (test monocularly if cross-fixating). The examiner should observe the presence of saccades to the right, left, up and down in both eyes. When looking for asymmetry (between vertical vs horizontal saccades and between left vs. right saccades) fixing on the bridge of the nose can aid the examiner.

### Interpreting some common clinical findings while testing saccades

- Saccadic latency in infants is up to 1 s for the primary saccade (200 ms in adults) and gross abnormalities are usually seen in SIF.
- SIF may be congenital with no recognisable pathology or associated with many underlying disorders. It is usually horizontal only and intermittent; if vertical it suggests a midbrain lesion or metabolic disorder such as Niemann–Pick disease type C or Gauchers disease type 3 [9].
- Isolated dysfunction of vertical saccades is due to a midbrain lesion such as haemorrhage/Niemann–Pick disease type C. It is usually caused by lesions of the riMLF which is the centre for vertical saccadic control. As upgaze movements are double innervated, abnormalities of downward saccades are often observed first.
- Lesions of the interstitial nucleus of Cajal also cause dysfunction of vertical saccades but usually in addition to a vertical gaze-evoked nystagmus (GEN).
- Lesions of the posterior commissure cause dysfunction of vertical saccades which may be accompanied by convergence-retraction nystagmus and lid retraction in parinaud dorsal midbrain syndrome. Pressure on the posterior commissure from raised intracranial pressure is also thought to give rise to the ‘setting-sun’ sign (eye seemingly fixed in downgaze) which is unique to infants and children [10].
- Lesions of the cerebellar oculomotor (dorsal) vermis or fastigial nucleus can cause saccadic dysmetria. Hypermetric saccades are seen in lesions of the fastigial nucleus (and Wallenberg syndrome, lateral medullary syndrome) and hypometric saccades with lesions of the dorsal vermis.
- Large visual field defects may also cause hypometric saccades into the area of loss and they are also seen in resolving DVM type 1.
- Isolated dysfunction (slowing ±hypometria) of horizontal saccades is due to pontine lesion of the PPRF, e.g., bleeding/glioma/Gaucher disease type 3.
- Square-wave jerks are to one side of fixation only (<5° from fixation), last 200 ms and are most commonly seen in spino cerebellar atrophies. They are an exaggeration of normal microsaccadic fixation movements and are seen in 90% of infants at up to 3/min and are rarely of key clinical significance.
- Opsoclonus is characterised by intermittent, multidirectional, back-to-back saccades and is best viewed after changing fixation or on lid closure. Ocular flutter describes the same anomaly but confined to horizontal eye movements. They share a common aetiology, which is thought to be loss of cerebellar inhibition of spontaneous saccades. Opsoclonus–myoclonus syndrome describes the combination of opsoclonus, myoclonus and ataxia and is most commonly associated with neuroblastoma, other para-neoplastic states and CNS infections [11]. Ocular flutter or hypermetric saccades can be seen as opsoclonus improves.
- Anti-saccades are voluntary saccades generated to the opposite direction of a target introduced into the peripheral visual field. They represent a voluntary saccade overcoming a reflexive saccade and may be misdirected (ie, implying a directional error) in children under seven, schizophrenia, ADHD and Tourette syndrome. They are rarely of clear diagnostic value in young children.
- ‘Voluntary nystagmus’ is an infrequent but persistent cause for referral in children. It is characterised clinically by back-to-back saccades (typically horizontal), usually induced by convergence effort and often accompanied by mild lid closure. It is therefore *not nystagmus* (as there are no slow phases). It can be simulated in many healthy subjects and clues to the diagnosis include: miosis, lid flutter and an inability to sustain the movements for more than 30–45 s.

## Gaze holding

### Underlying neuroanatomy

Gaze holding is controlled by a group of neural structures which differ for vertical/torsional and horizontal meridians. These distinct groups of structures are collectively termed the horizontal and vertical ‘neural integrators’. The horizontal neural integrator includes the nucleus prepositus hypoglossi (Pons), vestibular nuclei (Pons and Medulla) and vestibulo-cerebellum (Cerebellum). The vertical/torsional neural integrator is the Interstitial Nucleus of Cajal (Midbrain) although an intact flocculus (Cerebellum) is also necessary. Both neural integrators have multiple neural connections and their primary role is to coordinate tonic discharge to the extra-ocular muscles in order to maintain eccentric gaze.

### Clinical assessment of gaze holding

Observe the eyes in primary gaze and in vertical and horizontal eccentric gaze either by turning the patient’s head, or by presenting a large stimulus in eccentric view. Abnormalities of gaze holding are usually characterised by nystagmus, due to a slow drift towards primary position and a corrective saccade into the attempted position of eccentric gaze (GEN). Examination of nystagmus is detailed below.

### Interpreting some common clinical findings while examining gaze holding

- Typical signs of damage to the neural integrator, whether vertical, torsional or horizontal, include GEN (due to loss of eccentric gaze holding) and rebound nystagmus (nystagmus beats seen in the opposite direction to the eccentric gaze when the eye is returned to primary position). Abnormalities of VOR suppression (most commonly seen as failure of dampening post-rotational vestibular nystagmus following the spinning baby test) and OKR are also commonly seen together and reflect underlying cerebellar pathology.
- GEN can also be seen with the use of anti-convulsants, sedatives and other drug intoxications.
- GEN in all directions implies a cerebellar lesion, particularly the flocculus and has many causes.
- Isolated horizontal GEN is often caused by an isolated pontine lesion. This is because vestibulo-cerebellar disorders tend to yield additional oculomotor signs.
- Purely vertical GEN is due to a lesion of the vertical/torsional neural integrator which is the interstitial nucleus of Cajal (midbrain).
- The presence of rebound nystagmus may indicate chronic, rather than acute cerebellar disease.

## Vergence

### Underlying neuroanatomy

The neuroanatomy underlying vergence eye movements is poorly understood. Neurons within the mesenchephalic reticular formation (midbrain) and adjacent rostral superior colliculus have been identified as being related to vergence movements. These include neurons whose discharge is proportional to vergence angle (near response, NR neurons) and burst neurons. Subsets seem to exist for convergence and divergence activity but detail is currently lacking.

### Clinical assessment of vergence

Vergence movements are binocular co-ordinated movements in opposite directions in order to move the point of fixation towards, or away from the subject. Convergence movements can be observed in infants using a large visual target, moved slowly towards the nose in primary gaze from approximately two months of age. They are thought to be initially driven by accommodation but after 4 months they are driven by retinal disparity which coincides with the development of fusion and stereopsis [7]. From around 6 months, tests for fusional vergence include the 20 prism-dioptre base-out prism test and studies of fusional range. The examiner should note the range of vergence movements from a near point to a distance target.

### Interpreting some common clinical findings while testing vergence

- Vergence abnormalities are rare in children unless associated with strabismus.
- Neuropathology associated with isolated anomalies of vergence is very rare.
- Numerous reports and case studies have suggested that *convergence spasm* can occur in association with organic and non-organic disease. However, organic pathology is rarely found in an otherwise well child with isolated convergence spasm [12, 13].
- *Convergence insufficiency* describes a remote near point of convergence, variable exophoria for near and reduced fusional convergence at near. It is not associated with underlying intracranial lesions and when occurring in isolation, usually in adolescents, may be treated with orthoptic exercises and prisms [14–16].
- *Convergence paralysis* is characterised by complete loss of convergence and crossed diplopia. It is highly suggestive of intracranial pathology resulting from space occupying lesions of the midbrain, infection, trauma, demyelination or toxins [17–19]. It is therefore, usually associated with significant additional neurological signs and rarely encountered as an isolated phenomenon.
- *Divergence paralysis* is rare in both paediatric or adult practice. It is characterised by complete loss of divergence, esotropia for distance and normal convergence. Bilateral sixth nerve palsy should be excluded. Causes include intracranial infection and lesions of the brainstem and periaqueductal grey matter [20–22].
- *Divergence insufficiency* is a more controversial entity and is characterised by an esotropia for distance, normal convergence and variable or reduced fusional divergence [1]. It has been described as a continuum with divergence paralysis, in association with neuropathology including raised intracranial pressure or as a mechanical rather than neurological phenomenon [23–25]. In the absence of an acute presentation, other supranuclear eye movement anomalies or any neurological signs, underlying neuropathology is rare.

## Smooth pursuit

### Underlying neuroanatomy

The neuroanatomical pathways underpinning smooth pursuit movements have been studied extensively and many pathways and nuclei are known to play a part. The following is a simplification of the core circuit. Visual information from V1 is conveyed to area V5 (middle temporal and middle superior temporal areas) and then descends to the pontine nuclei. They are joined here by additional inputs from descending fibres from the frontal eye field (Brodman area 8). Fibres are relayed from the pontine nuclei to the flocculus and ventral paraflocculus of the cerebellum. Floccular Purkinje cells then encode eye position, velocity and to a smaller extent, acceleration. Projections then extend to the vestibular nuclei and on to the oculomotor nuclei within the brainstem [7, 26, 27].

### Clinical assessment of smooth pursuit

Testing should be performed both horizontally and vertically using a large, slow moving, brightly coloured target or a slowly rotating mirror. The examiner should observe the character of the smooth pursuit movements (jerky or smooth) and identify asymmetric smooth pursuit which can be aided by focussing on the bridge of the patient’s nose. An OKR drum or tape can be used if examining an uncooperative infant. Suppression of VOR and smooth pursuit are closely related oculomotor functions with similar underlying neurological substrates. Therefore, abnormalities in both systems commonly occur together. VOR suppression can be assessed by:

- Observing suppression of post-rotational nystagmus after the spinning baby test.
- Observing suppression of VOR while spinning baby and encouraging fixation on examiners face.
- Observing suppression of VOR using a target which moves with the head and encouraging horizontal and vertical head movements. The target can be the patients finger held in front of the face or a target on a tongue depressor held in the patient’s mouth.

### Interpreting some common findings while examining smooth pursuit

- Lesions of the flocculus or cerebellar pathways cause saccadic pursuit and loss of VOR suppression by visual fixation in addition to down beat nystagmus (DBN).
- Reduced smooth pursuit in all directions indicates a ‘general’ cerebellar flocculus disorder (drugs, alcohol, ataxias, etc.).
- Asymmetrical loss of smooth pursuit (i.e., reduced binocularly to a left vs. right moving target) suggests a structural lesion of the ipsilateral cortex or cerebellum. It may also indicate early-onset poor vision in one eye.

## Nystagmus

Nystagmus is a disorder of eye movement which can be defined as ‘a repetitive, to-and-fro movement of the eyes that is initiated by slow phases’ [7]. It is important to note that the pathology is the initial slow movement. This is followed by either a ‘corrective’ fast movement (jerk nystagmus) or a second slow movement (pendular nystagmus). It is relevant to many abnormalities of eye movement in children. Therefore, in the following section, we discuss the key clinical features to identify when nystagmus is found as part of the supranuclear eye movement examination described above.

Classification of nystagmus has been inconsistent in the literature and in the following section, we use the term infantile nystagmus syndrome (INS) and fusion maldevelopment nystagmus syndrome (manifest latent nystagmus or FMNS) according to the CEMAS classification of 2001 [28]. It is important for the clinician to note that patients with INS or FMNS may have a variety of underlying ocular/systemic conditions but these descriptions have some key eye movement signs which help to exclude (often more concerning) differential diagnoses.

### Clinical assessment of nystagmus

**Fig. 6:**
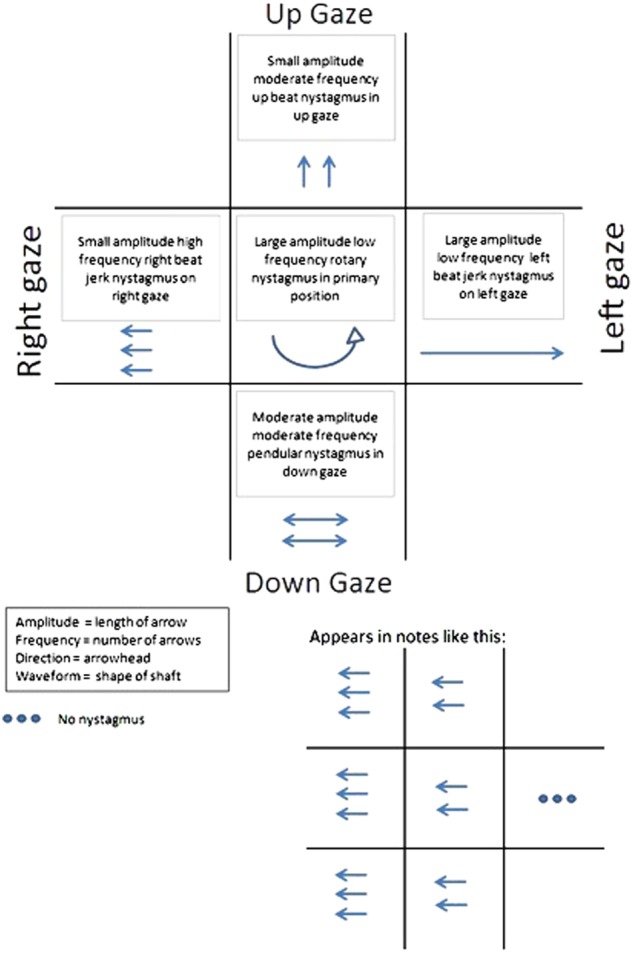
Recording nystagmus amplitude, frequency, direction and waveform in nine positions of gaze

- Observe the child at play to identify any consistent visual behaviours such as head positions and head shaking (often, but not always, after 1 year of age), in addition to signs of systemic neurological features such as ataxia.
- In addition to a routine, age-appropriate history, orthoptic examination and refraction, children’s eye movements should be observed in five positions of gaze and note taken of the direction of the fast phase of nystagmus (if jerk is seen), the amplitude of the nystagmus (fine or coarse) and the effect of monocular viewing and removal of fixation.
- Where MLN or FMNS is suspected, rotating the child to induce post-rotational nystagmus and then occluding each eye separately can make identification easier. The induced post-rotational nystagmus to the right or left will either add to the existing MLN or reduce it, depending on which eye is covered, yielding a clear inter-ocular difference in nystagmus intensity.
- Abnormal eye movements should be observed for at least 5 min (ideally 10) to examine for reversal of nystagmus direction and any alteration in head posture. Inconsistencies in history/examination/previous clinical notes may suggest an alternating head posture often due to periodic alternating nystagmus (PAN), which itself can be congenital or aquired.
- An examination of saccades/smooth pursuit/gaze holding/VOR/vergence should also be performed as described above.
- An ocular examination should be performed with the best age dependant equipment available specifically looking for signs of photophobia, retinal dystrophy, retinal/choroidal hypopigmentation, iris transillumination and anterior segment anomaly e.g., cataract.
- Ocular coherence tomography (OCT) of the macular should be performed where possible to identify foveal hypoplasia and other abnormalities of retinal morphology that would give an indication of retinal origin for the nystagmus.
- Where possible, electro-diagnostic tests should be performed including: an electroretinogram (ERG) and a visual evoked potential (VEP) to identify retinal and post-retinal pathology such as a retinal dystrophy, or abnormal decussation at the chiasm in albinism.
- Where possible, parents should be examined for the presence of subtle nystagmus, iris transillumination and foveal hypoplasia (seen in albinism phenotypes and *PAX6* gene disease) and retinal dystrophies (especially where equipment is not available to fully examine the young child and/or a family history is evident).
- Anomalous head postures are best viewed by asking the patient to view the smallest target they can resolve in the distance and giving them time to adopt a head posture if one is present.

- Videos of head postures and nystagmus can be extremely useful especially where only fleeting glimpses of the child are possible and to aid advice from other clinicians. In the authors’ practice, videos taken by parents or referring clinicians can provide the most useful part of any referral or request for advice.

- Documenting nystagmus can be achieved in a variety of ways. The following diagrammatic representation is used in the authors’ practice (Fig. 6).

## Interpreting some common clinical findings while examining nystagmus

### Infantile nystagmus syndrome (INS)

INS is characterised by horizontal nystagmus, staying horizontal in vertical gaze, associated with null zones, dampening with convergence and made worse by visual attention and stress. It is rarely associated with underlying neuropathology but can be seen due to congenital visual loss of any cause.

### Fusion maldevelopment nystagmus syndrome (FMNS, previously MLN)

FMNS is characterised by horizontal nystagmus which beats in the direction of the viewing eye, worsening in abduction and improving in adduction of the viewing eye. It is commonly seen with strabismus without neuropathology and is thought to be due to maldevelopment of fusion.

### Periodic alternating nystagmus (PAN)

PAN may be congenital (isolated or commonly seen in albinism) or related to identifiable lesions of the cerebellar nodulus/uvula. It alternates less regularly in congenital forms when it tends to be more asymmetrical and less predictable (asymmetrical periodic alternating nystagmus (APAN)).

### Vertical nystagmus

- DBN is often more pronounced in downgaze but also in horizontal gaze. It can be accompanied by an alternating skew mimicking a superior oblique over-action. Some children adopt a resulting chin down posture which can mimic tonic upgaze. DBN is typically caused by lesions of the cerebellar flocculus bilaterally (such as in Arnold–Chiari malformations) and can also be found less commonly in ‘idiopathic’ patients and those with retinal dystrophy.
- DBN may be responsive to treatment with aminopyridines and UBN and PAN also to Baclofen treatment.
- Vertical and multidirectional nystagmus can be caused by intracranial lesions/metabolic disease but also retinal dystrophies. We would advocate electro-diagnostic testing (where available without significant delay) prior to neuroimaging for this reason in children with apparently isolated vertical nystagmus.
- Up beat nystagmus (UBN) is usually due to lesions of the midbrain or medulla, e.g., tumours or multiple sclerosis in adults.
- See-saw nystagmus is characterised by a cyclic movement of both eyes. In one half of the cycle, one eye will rise and intort and the other eye will fall and extort; then, in the next half cycle, the vertical and torsional components are reversed. It is most commonly caused by parasellar masses but has been described in many other conditions. It is rare in children.
- Vertical nystagmus may be seen as a side effect from systemic medications, commonly antiepileptics, such as phenytoin.

### Vestibular nystagmus

Alexander’s law states that nystagmus intensity increases when the eyes are moved in the direction of the fast phase. Originally, described for peripheral vestibular nystagmus it also holds true for central vestibular nystagmus and possibly other forms. Torsional nystagmus is often vestibular and is rare in primary position when it is usually medullary in origin (Syringobulbia or Wallenberg syndrome).

## Some common clinical dilemmas

In this section, we discuss some of the more common clinical dilemmas faced by clinicians.

### How to differentiate end-point from GEN

This is a common dilemma. The following features help the clinician to differentiate end-point nystagmus from GEN. In the authors’ experience, using the following criteria, these patients rarely pose a significant diagnostic dilemma (Table 4).

**Table 4:** Differentiating end-point from gaze-evoked nystagmus

End-point nystagmus	Gaze-evoked nystagmus (GEN)
Unstained (dampens after a few beats)	Sustained. Continues after 20 s of eccentric fixation attempt
Delayed after the eccentric eye movement	Immediate on eccentric fixation
Usually only seen in extreme eccentric gaze	Often seen in only mildly eccentric gaze
Often low amplitude	Often high amplitude
Symmetrical	Often asymmetrical
No rebound nystagmus seen (nystagmus beating in the opposite direction once the eye has returned to its central position after 60 s eccentric gaze)	Rebound often seen
No other supranuclear eye movement abnormalities found.	Very rarely seen without co-existing smooth pursuit abnormalities
Typically horizontal nystagmus on side gazes only	May be horizontal and/or vertical

### How to differentiate FMNS or MLN from INS in a patient with early loss of fusion (such as strabismus or cataract)

This is a frequent question for clinicians and many cases will have some features of FMNS and other features suggesting INS. However, the most clinically relevant distinction to make is between those cases with INS/FMNS and those with additional features suggesting a neurological cause, requiring additional investigation and management.

- A full orthoptic examination looking for presence of early-onset manifest strabismus (which is often seen in FMNS) can provide a clue towards an FMNS diagnosis.
- A full examination of the anterior and posterior segments of the eye can identify monocular cataract or optic nerve hypoplasia leading to FMNS. Bilateral pathology may cause INS, FMNS or both.
- INS can include an FMNS component but is not always affected by monocular occlusion unlike isolated FMNS in which the direction of the fast phase always changes towards the viewing eye. INS with an FMNS component may result in an increase in frequency and amplitude with monocular occlusion, without a change in the fast phase direction.
- Null positions and head postures are frequent in INS and much less common in FMNS unless the child is cross-fixating. However, some children with very asymmetrical visual acuity and FMNS may demonstrate a head posture to utilise adduction to dampen the FMNS in the seeing eye.
- INS onset is often earlier (<6–12 months) than FMNS.

### How to differentiate opsoclonus/flutter and square-wave jerks from other eye movement conditions

Opsoclonus/flutter can occasionally be confused with nystagmus especially in younger children. Making the distinction is important for clinical management and a few key differences exist:

- Opsoclonus/flutter have characteristic, seemingly random, fast eye movements with no slow component (unlike nystagmus) and no inter-saccadic interval. When only horizontal they are described as flutter.
- They are often intermittent (unlike nystagmus) and induced by changes in fixation or blinking.
- Because opsoclonus/flutter is typically acquired and intermittent, children are often visibly troubled during periods of abnormal eye movement (unlike early-onset nystagmus).
- Onset of abnormal eye movements can be noted at any age in opsoclonus/flutter; however, for most children with INS, the abnormal eye movements are noted between 3 and 6 months of age and worsen before stabilising.
- Eye movement tics and voluntary nystagmus in older children has been confused with ocular flutter. However, as stated above, opsoclonus and ocular flutter are more common with changes in fixation or blinking and importantly, can be seen under closed eyelids which is rarely seen with tics and voluntary nystagmus.

- Pathological square-wave jerks are rarely seen in children but have been described in association with various underlying pathologies and medications. The key distinction is that they comprise of only fast (saccadic) movements rather than the anomalous slow movement seen in nystagmus.

### When to request neuroimaging in a child with an apparent isolated supranuclear eye movement abnormality?

For most clinicians, this is a major concern when faced with a child who has (often striking) abnormal eye movements. Practice varies widely and is often governed by access to clinical tools, equipment, necessity of general anaesthesia for brain imaging studies and varying opinion on risk of general anaesthesia in young children. The following is a guide based on the authors own experience and practice.

- In most tertiary referral practice, children with clinical features consistent with INS (conjugate, clinically horizontal nystagmus, staying horizontal in vertical gaze, ±null zone, onset between 3 and 6 months of age, no other anomaly of VOR/SP/Vergence/vertical OKR and accelerating exponential slow phases on eye movement recordings if available), otherwise normal neurological development, normal posterior segment examination and ERG/VEP would not undergo neuroimaging in the first instance. Should subsequent examinations yield other eye movement findings, additional developmental issues or new ophthalmic findings (such as subtle optic nerve hypoplasia) neuroimaging would be sought. A similar approach would be taken in a child with typical FMNS (MLN) and early manifest strabismus or either INS or FMNS in a child with an underlying condition known to be associated with nystagmus such as Down’s syndrome.
- Where a clear underlying cause for the nystagmus is identified through clinical examination, OCT and ERG/VEP, such as early-onset retinal dystrophy or features of albinism, neuroimaging would not be initially sought.
- Unilateral nystagmus (or significant inter-ocular asymmetry) or supranuclear eye movement anomalies besides nystagmus would be joint managed with paediatric neurologists and neuroimaging would form a part of a broader set of urgent investigations in all cases.

## Summary

Isolated abnormalities of specific supranuclear eye movements in children are rare. For most, a pattern will emerge from gaining some information about each of the systems described above, in addition to an orthoptic workup, refraction, good history, electro-diagnostic tests (where available), examination of the family and basic neurological examination. FMNS (MLN) is common, as is INS related to albinism or an eventual ‘idiopathic’ diagnosis. Additional information can be gleaned from eye movement recordings and hand-held OCT devices but in many cases the clinical diagnosis does not rely solely on these scarce resources. Similarly, the role of genetic testing in these patients is evolving but at present, hampered by the resources, financing and infrastructure required for sequencing and variant interpretation. It is likely that in coming years these technologies will become a part of routine clinical practice but even then, a methodical and detailed clinical examination will remain paramount in assessing and managing this complex group of patients.

## References

[1] Cassidy L, Taylor D, Harris C (2000). Abnormal supranuclear eye movements in the child: a practical guide to examination and interpretation. Surv Ophthalmol.

[2] Bronstein AM, Patel M, Arshad Q (2015). A brief review of the clinical anatomy of the vestibular-ocular connections—how much do we know?. Eye.

[3] Shawkat FS, Kingsley D, Kendall B, Russell-Eggitt I, Taylor DSI, Harris CM (1995). Neuroradiological and eye movement correlates in children with intermittent saccade failure. Neuropediatrics.

[4] Casteels I, Harris CM, Shawkat F, Taylor D (1992). Nystagmus in infancy. Br J Ophthalmol.

[5] Admiraal RJ, Huygen PL (1997). Vestibular areflexia as a cause of delayed motor skill development in children with the CHARGE association. Int J Pediatr Otorhinolaryngol.

[6] Konrádsson K, Magnusson M, Andréasson S (1998). Perform vestibular test among all small deaf children! Early detection of Usher syndrome improves the possibilities of communication in the event of later deaf-blindness. Lakartidningen.

[7] Leigh R. John, Zee David S. (2015). Vergence Eye Movements. The Neurology of Eye Movements.

[8] Russell-Eggitt I, Harris CM, Kriss A (1998). Delayed visual maturation: an update. Dev Med Child Neurol.

[9] Shawkat FS, Kingsley D, Kendall B, Russell-Eggitt I, Taylor DS, Harris CM (1995). Neuroradiological and eye movement correlates in children with intermittent saccade failure: “ocular motor apraxia”. Neuropediatrics.

[10] Brodsky M. Pediatric neuro-ophthalmology. Second edition. Springer; 2010 https://www.springer.com/gb/book/9780387690698.

[11] Blaes F, Dharmalingam B (2016). Childhood opsoclonus–myoclonus syndrome: diagnosis and treatment. Expert Rev Neurother.

[12] Fekete R, Baizabal-Carvallo JF, Ha AD, Davidson A, Jankovic J (2012). Convergence spasm in conversion disorders: prevalence in psychogenic and other movement disorders compared with controls. J Neurol Neurosurg Psychiatry.

[13] Ghosh A, Padhy SK, Gupta G, Goyal MK (2014). Functional convergence spasm. Indian J Psychol Med.

[14] Lavrich JB (2010). Convergence insufficiency and its current treatment. Curr Opin Ophthalmol.

[15] Davies CE (1946). Orthoptic treatment of convergence insufficiency. Arch Ophthal.

[16] McGregor ML (2014). Convergence insufficiency and vision therapy. Pediatr Clin North Am.

[17] Gilad E, Biger Y (1986). Paralysis of convergence caused by mushroom poisoning. Am J Ophthalmol.

[18] Wylie J, Campbell C, Pope J, Akikusa J, Laxer RM, Nicolle D (2006). Convergence paralysis as a manifestation of polyarteritis nodosa. Can J Neurol Sci.

[19] Day C, Arora S, Adam RS, Rodriguez AR (2011). Idiopathic convergence paralysis: a rare case. Am Orthopt J.

[20] Tsuda H, Shinozaki Y, Tanaka K, Ohashi K (2012). Divergence paralysis caused by acute midbrain infarction. Intern Med.

[21] Bakker SL, Gan IM (2008). Temporary divergence paralysis in viral meningitis. J Neuroophthalmol.

[22] Zentmayer W (1913). Report of a case of paralysis of divergence. Trans Am Ophthalmol Soc.

[23] White JW (1947). Divergence insufficiency and paralysis. N Y State J Med.

[24] Mittelman D (2013). Divergence insufficiency esotropia is a misnomer. JAMA Ophthalmol.

[25] Kang HM, Kim HY (2011). A case of pediatric idiopathic intracranial hypertension presenting with divergence insufficiency. Korean J Ophthalmol.

[26] Leung HC, Suh M, Kettner RE (2000). Cerebellar flocculus and paraflocculus Purkinje cell activity during circular pursuit in monkey. J Neurophysiol.

[27] Cullen KaVH, M. Brainstem pathways and premotor control; 2013.

[28] Workshop C. Classification of Eye Movement Abnormalities and Strabismus (CEMAS) Workshop report. National Eye Institute Sponsored Workshop; 2001.

[29] Bielecki J, Hoeg JT, Garm A (2013). Fixational eye movements in the earliest stage of metazoan evolution. PLoS ONE.

[30] Walls G (1962). The evolutionary history of eye movements. Vision Res.

[31] Self J, Mercer C, Boon EM, Murugavel M, Shawkat F, Hammans S, et al. Infantile nystagmus and late onset ataxia associated with a CACNA1A mutation in the intracellular loop between s4 and s5 of domain 3. Eye; 2009. 10.1038/eye.2008.389 19182766

[32] Bronstein A, Patel M, Arshad Q (2015). A brief review of the clinical anatomy of the vestibular-ocular connections—how much do we know?. Eye.

[33] Cordero L, Clark DL, Urrutia JG (1983). Post-rotatory nystagmus in the full-term and premature infant. Int J Pediatr Otorhinolaryngol.

[34] Valmaggia C, Rütsche A, Baumann A, Pieh C, Bellaiche Shavit Y, Proudlock F (2004). Age related change of optokinetic nystagmus in healthy subjects: a study from infancy to senescence. Br J Ophthalmol.

